# Genetic, serological and biochemical characterization of *Leishmania tropica* from foci in northern Palestine and discovery of zymodeme MON-307

**DOI:** 10.1186/1756-3305-5-121

**Published:** 2012-06-18

**Authors:** Kifaya Azmi, Lionel Schnur, Gabriele Schonian, Abedelmajeed Nasereddin, Francine Pratlong, Fouad El Baidouri, Christophe Ravel, Jean-Pierre Dedet, Suheir Ereqat, Ziad Abdeen

**Affiliations:** 1 Al-Quds Nutrition and Health Research Center, Faculty of Medicine, Al-Quds University, Abu-Deis, P.O. Box: 20760, West Bank, Palestine; 2 Kuvin Centre for the Study of Infectious and Tropical Diseases, IMRIC, Hebrew University-Hadassah Medical School, Jerusalem, Israel; 3 Institute of Microbiology and Hygiene, Charité University Medicine Berlin, Dorotheenstr. 96, Berlin, D-10098, Germany; 4 Université Montpellier 1, Centre National de référence des Leishmania, UMR MIVEGEC (UM1, CNRS 5290, IRD224). Laboratoire de Parasitologie-Mycologie, CHU de Montpellier, 39, Avenue Charles Flahault, Montpellier Cedex 5, 34295, France

## Abstract

**Background:**

Many cases of cutaneous leishmaniasis (CL) have been recorded in the Jenin District based on their clinical appearance. Here, their parasites have been characterized in depth.

**Methods:**

Leishmanial parasites isolated from 12 human cases of CL from the Jenin District were cultured as promastigotes, whose DNA was extracted. The ITS1 sequence and the 7SL RNA gene were analysed as was the kinetoplast minicircle DNA (kDNA) sequence. Excreted factor (EF) serotyping and multilocus enzyme electrophoresis (MLEE) were also applied.

**Results:**

This extensive characterization identified the strains as *Leishmania tropica* of two very distinct sub-types that parallel the two sub-groups discerned by multilocus microsatellite typing (MLMT) done previously. A high degree of congruity was displayed among the results generated by the different analytical methods that had examined various cellular components and exposed intra-specific heterogeneity among the 12 strains.

Three of the ten strains subjected to MLEE constituted a new zymodeme, zymodeme MON-307, and seven belonged to the known zymodeme MON-137. Ten of the 15 enzymes in the profile of zymodeme MON-307 displayed different electrophoretic mobilities compared with the enzyme profile of the zymodeme MON-137. The closest profile to that of zymodeme MON-307 was that of the zymodeme MON-76 known from Syria.

Strains of the zymodeme MON-307 were EF sub-serotype A_2_ and those of the zymodeme MON-137 were either A_9_ or A_9_B_4_. The sub-serotype B_4_ component appears, so far, to be unique to some strains of *L. tropica* of zymodeme MON-137. Strains of the zymodeme MON-137 displayed a distinctive fragment of 417 bp that was absent in those of zymodeme MON-307 when their kDNA was digested with the endonuclease RsaI. kDNA-RFLP after digestion with the endonuclease MboI facilitated a further level of differentiation that partially coincided with the geographical distribution of the human cases from which the strains came.

**Conclusions:**

The Palestinian strains that were assigned to different genetic groups differed in their MLEE profiles and their EF types. A new zymodeme, zymodeme MON-307 was discovered that seems to be unique to the northern part of the Palestinian West Bank. What seemed to be a straight forward classical situation of *L. tropica* causing anthroponotic CL in the Jenin District might be a more complex situation, owing to the presence of two separate sub-types of *L. tropica* that, possibly, indicates two separate transmission cycles involving two separate types of phlebotomine sand fly vector.

## Background

**Figure 1:**
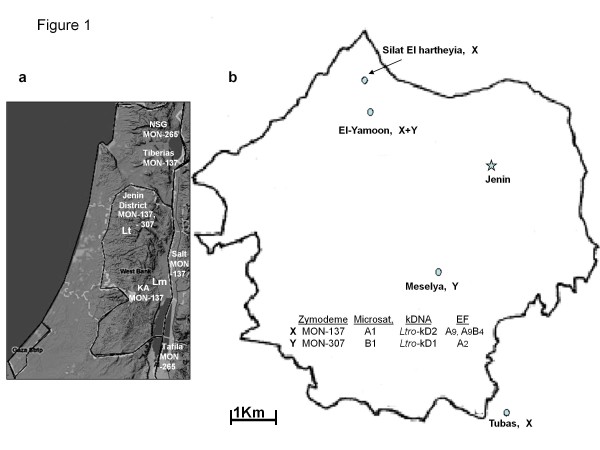
**Map of the West Bank and Jenin District. a**, map of the West Bank and adjacent areas mentioned in the text, showing the distribution of the zymodemes to which the two reference strains of *L. tropica*, ISER/IL/98/LRC-L758; MHOM/IL/97/P963, belonged and of the zymodeme MON-265; KA = Kfar Adumim; NSG = the focus just north of the Sea of Galilee; Lt = foci of *L. tropica*; Lm = foci of *L. major* ; **b**, map of the Jenin District, showing the foci from which the 12 cases of CL came and the distribution of the two types of *L. tropica* isolated from the cases, designated X and Y in the map to correspond with their characteristic biochemical (zymodemal affiliation), genetic (microsatellite affiliation and kDNA type) and serological (excreted factor (EF) serotype) profiles.

*Leishmania major* and *L. tropica* cause cutaneous leishmaniasis (CL) in Palestine, and *L. infantum* has been known to cause CL without the clinical manifestation of VL (unpublished data from Palestine). CL caused by *L. infantum* was also confirmed in the Mediterranean region [1]. Cases caused by *L. tropica* seem to occur mainly in the northern regions [2,3] and those caused by *L. major* mainly in the lower Jordan Valley in the vicinity of Jericho [4-6] (Figure 1)a. Often they exist in close proximity despite differences in their specific biotopes and general epidemiology. Classically, *L. major* was and is considered to be zoonotic, involving many different species of rodents as the animal reservoir in different Old World foci, and *L. tropica* was considered to be purely anthroponotic with direct transmission from person to person. However, more recently it has become clear that in some cases *L. tropica* is zoonotic [7]. Since people travel more widely than the sand fly vectors and reservoir animals that delineate foci of these species, it is difficult to know with certainty where human subjects contracted their CL. The clinical appearance of lesions is not indicative of the aetiology and characterization and identification of the pathogen are an integral part of the diagnosis of human CL. A single effective method often suffices for the diagnosis of an infectious disease like leishmaniasis, but several species of *Leishmania*, causing different clinical spectra, infect humans and these must be clearly identified and differentiated. A broader characterization incorporating several different methods is more useful in achieving this and exposing variation among the infectious agents. This might be significant in prognosis and the need of specific treatment of cases. A broader characterization is also important when what appear to be leishmanial parasites are isolated from sand flies and animals that might prove to be the vectors and reservoirs, thus, elucidating the epidemiology. *L. tropica* is a very heterogeneous species and broad characterization of its strains assumes greater importance. All of this applies to the situation existing in Palestine. The following molecular biological characterization indicates genetic variation among strains of *L. tropica*, EF serotyping indicates antigenic variation and MLEE indicates biochemical variation between enzymes fulfilling the same function.

**Figure 2:**
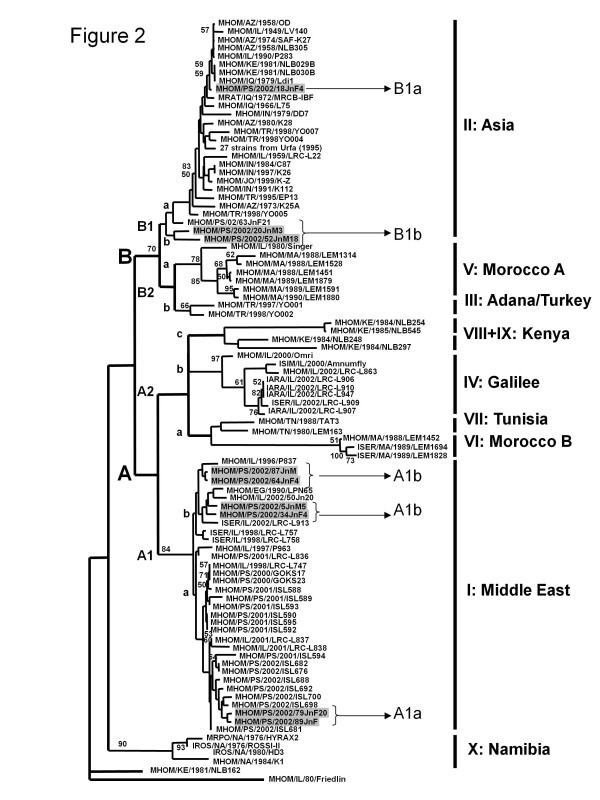
**Neighbour-joining dendrogram of strains of* Leishmania tropica.*** Modification of the neighbour-joining dendrogram of strains of *Leishmania tropica* in Figure 3 in Schwenkenbecher [3], indicating the derivation of the microsatellite subgroup designations. Note, three errors in Figure 3 in Schwenkenbecher [3] have been corrected here. In microsatellite cluster II: Asia, MHOM/PS/2002/18JnF8 should have been MHOM/PS/2002/18JnF4 and MHOM/PS/2002/52JnF21 should have been MHOM/PS/2002/63JnF21, and in microsatellite cluster I: Middle East the WHO code MHOM/IL/1997/P963 appears in two places. The WHO code of the one on the upper edge of the cluster should have been MHOM/IL/1996/P837.

Schwenkenbecher [3] used 21 microsatellite markers to determine the profiles of 117 strains of *Leishmania tropica* which separated into ten genetic clusters I to X. Of the 27 strains that came from Palestinian foci, 9 were isolated from CL cases living in the Jenin District (Figure 1)b. These grouped in the clusters ' I: Middle East ' and ' II: Asia ' whereas strains from other Palestinian foci, e.g., the Jericho area and Samaria, were all assigned to the cluster 'I: Middle East' (Figure 2). To understand why two different genetic groups of *L. tropica* exist in a small area, genotypic differences were compared with phenotypic ones. Since MLMT using 21 microsatellite markers is both time- and labour-consuming, simpler and quicker methods for genotyping strains were developed. For this, nine of the previously characterized strains from the Jenin District, representing different genotypes assigned to the different genetic clusters and three strains newly isolated from CL patients from there, were selected for further molecular biological (kinetoplast minicircle DNA analysis), serological (excreted factor (EF) serotyping) and biochemical (multilocus enzyme electrophoresis-MLEE) characterization. Although *L. tropica* has been known to exist in Palestine for many years and many strains of it have been isolated and characterized in various ways, none had been characterized by MLEE and assigned to zymodemes. This study exposed substantial genetic, antigenic and biochemical variation among strains of *L. tropica* isolated from Palestinian human cases of CL that included the discovery of a new zymodeme of the species, i.e., zymodeme MON-307. The strains studied came from the Jenin District where the average annual incidence of human CL is 23 cases per 100,000 (Azmi, unpublished data).

## Methods

### Isolation and cultivation of leishmanial parasites and extraction of their DNA

Twelve patients presenting cutaneous lesions suspected of being CL were referred to clinics of The Ministry of Health in the City of Jenin for diagnosis and treatment. None had travelled abroad. Seven had a single lesion on the face, four had a single lesion on the hand and one had a lesion on the hand and also one on the leg. A single isolate was cultured from each case. All were treated with Pentostam before identification of their strains and the outcome of treatment is unknown. Promastigotes were grown in culture from infected skin tissue aspirates as described by Azmi [8]. Table 1 lists the 12 strains isolated and gives their geographical origin. It also lists two Israeli reference strains of *L. tropica*, ISER/IL/98/LRC-L758 and MHOM/IL/97/P963 (= LRC-L725) and one of *L. major*, MHOM/IL/67/JerichoII (=LRC-L137), for comparison in identifying the strains.

**Table 1:** **The strains of*Leishmania*used in this study**

**WHO code**	**LRC#**	**Geographic origin**	**Microsatellite**		**Clade kDNA^b^**	**EFserotype^c^**	**Zymodeme^d^**
			**Cluster^a^**	**Subgp**			
MHOM/PS/2002/18JnF4*	L891	Meselya	II	B1a	A *Ltro*-kD1/3	A2	MON-307
MHOM/PS/2002/20JnM3*	L884	El-Yamoon	II	B1b	A *Ltro*-kD1/2	A2	MON-307
MHOM/PS/2002/52JnM18*	L890	El-Yamoon	II	B1b	A *Ltro*-kD1/1	A2	MON-307
MHOM/PS/2002/5JnF5*	L893	El-Yamoon	I	A1b	B *Ltro*-kD2/2	A9	MON-137
MHOM/PS/2002/87JnM*	L887	El-Yamoon	I	A1b	B *Ltro*-kD2/5	A9B4	MON-137
MHOM/IL/1997/P963*	L725	Tiberias, Israel	I	A1a	B *Ltro*-kD2/1	A9B4	MON-137
MHOM/PS/2002/79JnF20*	L885	Silat El hartheyia	I	A1a	B *Ltro*-kD2/6	A9B4	MON-137
MHOM/PS/2002/89JnF*	L886	Silat El hartheyia	I	A1a	B *Ltro*-kD2/6	A9B4	MON-137
MHOM/PS/2002/64JnF4*	L889	Silat El hartheyia	I	A1b	B *Ltro*-kD2/6	A9B4	MON-137
MHOM/PS/2002/34JnF4*	L882	Silat El hartheyia	I	A1b	B *Ltro*-kD2/3	A9B4	MON-137
ISER/IL/1998/LRC-L758*	L758	Kfar Adumim, Israel	I	A1b	B *Ltro*-kD2/1	A9B4	MON-137
MHOM/PS/2002/31JnM17	L883	Tubas	nd		B *Ltro*-kD2/6	A9B4	MON-137
MHOM/PS/2008/336JnM71	L1324	Silat El hartheyia	nd		B *Ltro*-kD2/4	A9B4	nd
MHOM/PS/2002/35JnF45	L888	Jenin City	nd		B *Ltro*-kD2/6	A9	nd
MHOM/IL/1967/JerichoII	L137	Near Jericho	Lmj 39		Lmaj-kD1	A1	MON-26

* Strains previously analysed by multilocus microsatellite typing (MLMT) using 21 microsatellite markers from Schwenkenbecher [3]^a^, their microsatellite cluster (I and II) , Subgroup (Subgp) as delineated here (Figure 2); ^b^, kDNA PCR-RFLP; ^c^, excreted factor (EF) serotype done according to Schnur and Zuckerman [15] ; ^d^, MLEE done according to Rioux [18] and Pratlong [19] ), and of *L. major* MOHM/IL/1967/JerichoII (=LRC-L137) using 10 microsatellite markers and taken from Al Jawabreh [39]. The Israeli strains of *L. tropica* LRC-L725 and LRC-L758 were used as reference strains of *L. tropica*; nd, not done.

Mass cultures were grown in Schneider’s Drosophila medium supplemented with 10% heat inactivated FCS with 2 mM L-glutamine, containing penicillin at 200 IU/ml and streptomycin at 200 μg/ml. Parasite DNA was extracted using the high pure PCR template purification kit (Roche Diagnostics GmbH, Mannheim, Germany).

### Strain characterization

#### Identification of leishmanial species

The three strains LRC-L883, -L1324 and –L888 were identified here by amplification and restriction fragment length polymorphism (RFLP) analysis of either the ITS1 sequence or a partial 7SL RNA gene as described previously [9,10]. The other nine Palestinian strains had been identified and characterized previously by ITS1 [9] and 7SL [10] and also MLMT analysis, which uses microsatellites that are specific for the species *L. tropica*[3]. DNA from the reference strains *L. infantum* MHOM/TN/80/IPT1 and *L. major* MHOM/TM/73/5ASKH, and from two local strains of *L. tropica*, ISER/IL/98/LRC-L758 and MOHM/IL/97/P963 (=LRC-L725), all obtained from the WHO Reference Center for the Leishmaniases, the Hebrew University-Hadassah Medical School, Jerusalem, were amplified in each reaction as positive controls. The inclusion of control samples from *L. major* and *L. infantum* was considered necessary as strains of these two species also circulate in the overall geographical region where *L. major* also causes CL in humans and *L. infantum*, though mainly associated with human cases of infantile VL, has been known to cause CL without being associated with patent visceral manifestations [1,11]. Corresponding preparations of reaction buffer without leishmanial DNA were included as negative controls.

#### Restriction fragment length polymorphism (RFLP) analysis of the kinetoplast minicircle DNA (kDNA)

PCR amplification of the leishmanial kDNA minicircle sequence from cultured promastigotes, was done according to Anders [12], using the primer pair Uni 21 (5’-GGG GTT GGT GTA AAA TAG GCC) and Lmj4 (5’-CTA GTT TCC CGC CTC CGA G) based on a minicircle sequence of *L. major*[13] and applying the PCR conditions described by Schnur [14]. The PCR product was digested separately with either the endonuclease *Rsa*I or *Mbo*I (Promega, Madison, WI), according to the manufacturer’s instructions. kDNA of *L. tropica* ISER/IL/98/LRC-L758 was included as a positive reference and that of *L. major* MHOM/IL/67/JerichoII (=LRC-L137) as the out-group. kDNA-RFLP results were analyzed using the RAPDistance Package version 1.04 (http://www.anu.edu.au/Bozo/software/). All the fragments seen, represented by bands, were numbered and scored: 0 for the absence of a band; 1 for its presence. Whether fragments were identical or similar was measured according to the overall profile seen after digestion with each endonuclease and the number of shared bands among each enzyme's profiles. A dendrogram was constructed based on the combined fragmentation of the restriction enzymes *Rsa*I and *Mbo*I, using the distance matrix (Neighbor joining) option. These are not bootstrap values. The values are Jaccard distance computed as binary Jaccard, which represents the measure of dissimilarity.

#### Excreted factor (EF) serotyping

This was done according to Schnur and Zuckerman [15], using standard polyclonal anti-leishmanial serotyping sera raised in rabbits and known reference EFs for comparisons and the designation of serotypes and sub-serotypes [16]. These correlate, in most cases, with leishmanial species definitions based on other distinguishing criteria. The standard serotyping sera were: 1) anti-*L. tropica* LRC-L36, which reacts only with EFs of the serotype A and, through partial cross-reactivities, also permits the designation of serotype A sub-serotypes; 2) anti-*L. donovani,* LRC-L52, which reacts only with EFs of the serotype B and differentiates the sub-serotypes B2 and B4. The reference EFs were those from the strains: 1) *L. tropica* LRC-L36 from Baghdad, Iraq, that indicates the sub-serotype A_2_, specific for one of the two known serological variants of *L. tropica*; 2) *L. tropica* LRC-L682 from Sanliurfa, Turkey, that indicates the sub-serotype A_9_, specific to the other serological variant of *L. tropica*; 3) *L. major* LRC-L137 from the Jordan Valley, Israel, that indicates the sub-serotype A_1;_ 4) *L. donovani* LRC-L133 from, Humera, Ethiopia, that indicates the sub-serotype B_2_, associated with the species *L. donovani and L. infantum*.

#### Multilocus enzyme electrophoresis (MLEE)

MLEE was done according to Maazoun [17] using thick starch gel electrophoresis to study the 15 enzymes listed by Rioux [18]: Malate dehydrogenase (MDH) E.C. 1.1.1.37; Malic enzyme (ME) E.C. 1.1.1.40; Isocitrate dehydrogenase (ICD) E.C. 1.1.1.42; Phosphogluconate dehydrogenase (PGD) E.C. 1.1.1.44; Glucose-6-phosphate dehydrogenase (G6PD) E.C. 1.1.1.49; Glutamate dehydrogenase (GLUD) E.C. 1.4.1.3; Diaphorase NADH (DIA) E.C. 1.6.2.2; Nucleoside purine phosphorylase 1 (NP1) E.C. 2.4.2.1; Nucleoside purine phosphorylase 2 (NP2) E.C. 2.4.2.2; Glutamate-oxaloacetate transaminase 1 (GOT1) E.C. 2.6.1.1; Glutamate-oxaloacetate transaminase 2 (GOT2) E.C. 2.6.1.1; Phosphoglucomutase (PGM) E.C. 5.4.2.2; Fumarate hydratase (FH) E.C. 4.2.1.2; Mannose phosphate isomerase (MPI) E.C. 5.3.1.8; Glucose phosphate isomerase (GPI) E.C. 5.3.1.9.). Two Israeli strains of *L. tropica* (ISER/IL/98/LRC-L758 and MHOM/IL/97/P963 = LRC-L725) belonging to the zymodeme MON-137 and one of *L. major* (MHOM/IL/67/JerichoII = LRC-L137) belonging to the zymodeme MON-26 from areas close to the Palestinian foci from where the Palestinian strains came from were included for comparison.

Pratlong [19] applied phenetic analysis to the enzyme profiles of 1,048 strains isolated from human cases of CL, animals hosts and sand flies from many Old World foci and revealed the interrelationship of enzyme profiles of the strains of *L. tropicaL. aethiopica* and *L. major*. The different types of zymodeme encompassed by the species *L. tropica* fell into four sub-groups: (a); (b); (c); (d). With the discovery of the new zymodeme MON-307 of *L. tropica*, as of this study, the branch containing the zymodemes of the strains of *L. tropica* was reconstructed to include zymodeme MON-307 and, also, a second zymodeme encompassing an Israeli strain of *L. tropica* from the Negev [14] to determine to which other zymodemes they were closely related and insert them into their correct zymodemal sub-groups.

#### Taxonomical methods

Phenetic analysis was based on 15 isoenzyme loci, 39 zymodemes and 165 characters (electromorphs). Of the 39 zymodemes of *L. tropica*, 37 are listed in Pratlong [19] and two were the new zymodemes MON-288 from Israel and MON-307 from Palestine. On the hypothesis that *Leishmania* is ‘mainly’ diploid [20], multiband patterns were considered to be heterozygous and electromorph values were duplicated. Unweighted Pair Group Method with Arithmetic mean (UPGMA), Neighbour Joining (NJ) and Wagner analysis were used to cluster the stocks. Robustness of the nodes was statistically tested by bootstrap analysis, using the phylip3.6 package [21].

## Results

### Isolation and species identification of leishmanial parasites from suspect cases of CL

All 12 patients presenting cutaneous lesions were diagnosed as cases of CL and parasites were isolated from all of them (Table 1).

Amplification of the 300 bp ITS1 sequence and the 185 bp 7SL RNA gene gave the same-sized products and RFLP patterns, respectively, for all 12 Palestinian strains and the two Israeli reference strains of *L. tropica* (ISER/IL/98/LRC-L758 and MHOM/IL/97/P963 = LRC-L725). These differed from those of the reference strains of *L. infantum* (MHOM/TN/80/IPT1) and *L. major* (MHOM/TM/73/5ASKH) (data not shown).

### Characterization of strains of *L. tropica*

#### PCR-RFLP analysis of kDNA

**Figure 3:**
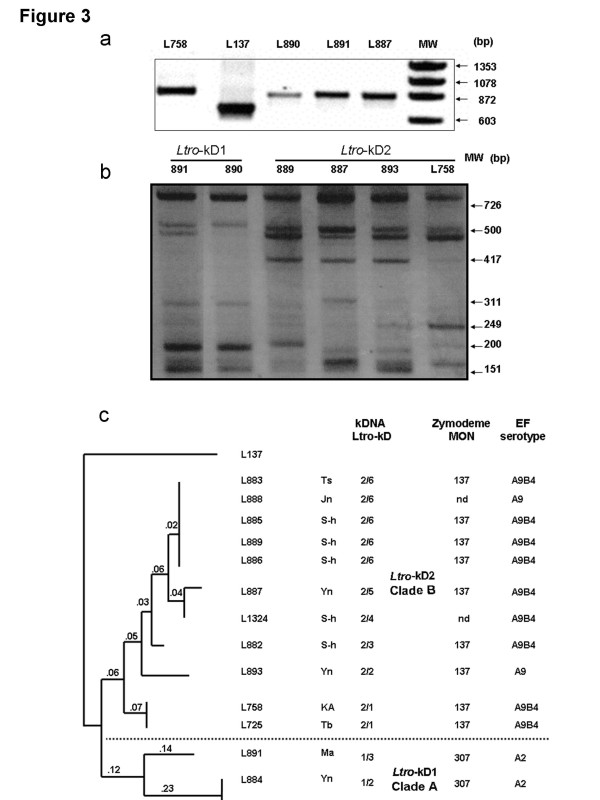
**The kDNA PCR and RFLP products of the 12 Palestinian* L. tropica*strains. a**, The kDNA PCR products of three of the 12 Palestinian strains: LRC-L890, -L891, -L887, and reference strains of *L. tropica*, -L758, and *L. major*, -L137; MW: molecular weight marker ΦX174 DNA/Hinf; **b**, The RFLP patterns of the six different genotypes resulting from digestion of kDNA PCR products with *Rsa*I. MW: molecular weight marker ΦX174 DNA/HaeIII Markers; **c**, Dendrogram based on the kDNA restriction fragment length polymorphism (RFLP) of the strains of *L. tropica* after digestion of their PCR products, separately, with the endonucleases *Rsa*I and *Mbo*I, which yielded ten sub-genotypes that segregated into the Clade A, with kDNA sub-type *Ltro*-kD1 and the Clade B, with kDNA sub-type *Ltro*-kD2. It was constructed using RAPDistance Package (version 1.04) and was based on the presence or absence of the bands in each sample. The numbers on the branches represent the % of difference. The lengths of the branches indicate the degree of similarity in fingerprints among the strains of *L. tropica* relative to the strain of *L. major*, MHOM/IL/67/JerichoII (=LRC-L137), which served as an out-group in this analysis: Ts = Tubas; Ma = Meselya; Yn = El-Yamoon; Jn = Jenin; S-h = Silat El hartheyia; Tb = Tiberias; KA = Kfar Adumim. The zymodemal designations and EF serotypes are given for complete comparison.

Amplification of the kDNA minicircle sequence of the 12 Palestinian and two Israeli reference strains of *L. tropica*, (ISER/IL/98/LRC-L758 and MHOM/IL/97/P963 = LRC-L725), gave the same-sized (872 bp) PCR product (Figure 3)a, which differed from the smaller-sized (650 bp) PCR product of *L. major* (MHOHM/IL/67/JerichoII = LRC-L137). RFLP analysis of all fourteen PCR products from the strains of *L. tropica* after their digestion with RsaI did produce different kDNA RFLP profiles that consisted of seven to nine bands ranging in size from 800 bp to 150 bp but of two basic kinds: *Ltro*-kD1 and *Ltro*-kD2, that differed mainly by the presence of a 417 bp component in only the *Ltro*-kD2 profiles (Figure 3)b. These two types of profile were reproducible in different gels and separated the strains into two clusters (Table 1 and Figure 3b and 3c). Digestion of the PCR products from the strains of *L. tropica* with MboI also produced different kDNA RFLP profiles, consisting of three to nine bands that were of two basic kinds and congruent with the separation seen for digestion with RsaI. The RAPDistance Package version 1.04 (http://www.anu.edu.au/Bozo/software/) used to analyze the kDNA RFLP fragments seen in the profiles after digestion separately with RsaI and MboI exposed nine different genetic variants, three, i.e., *Ltro*-kD1/1-3, falling into kDNA Clade A, and six, i. e., *Ltro*-kD2/1-6, falling into kDNA Clade B (Table 1 and Figure 3c). It is not surprising that the distances shown in the dendrogram are 'low' since the strains are all strains of a single species, *L. tropica*. However, the distance is greater between the two sub-groups of *L. tropica* seen in the dendrogram, and the distance between both sub-groups of *L. tropica* and the strain of *L. major* used as the out-group is much greater.

#### Excreted factor (EF) serotyping

**Figure 4:**
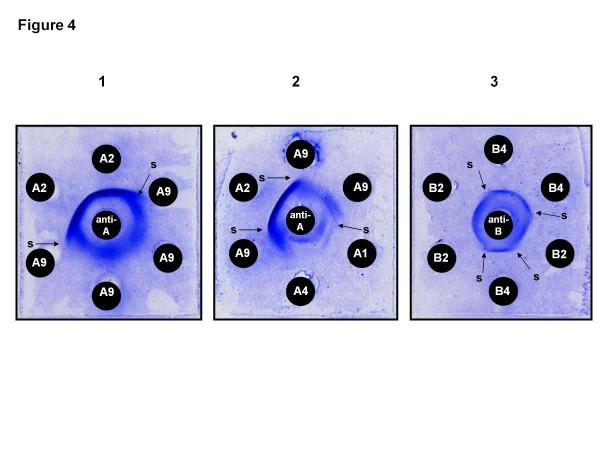
**Leishmanial excreted factor (EF)-antibody precipitation patterns.** Leishmanial excreted factor (EF)-antibody precipitation patterns between serotype A and serotype B anti-leishmanial specific sera and serotype A and serotype B EFs of different sub-serotype: 1 and 2, EF sub-serotype A reactions; 3, EF sub-serotype B reactions. Serotype A EFs react only with anti-serotype A sera. Serotype B EFs react only with anti-serotype B sera. EFs that react with, both, anti-serotype A and anti-serotype B sera are of mixed AB serotype where both the A and the B component can be of different sub-serotype, giving various mixed AB sub-serotypes, e. g., A_9_B_4_ as in some of the strains of *L. tropica* studied. Variation in the style of banding patterns and the formation of spurs (s) at the junctions of adjacent precipitation bands indicate incomplete identity of EFs of the same serotype, relegating them to different sub-serotypes of the serotype. EFs of the sub-serotypes A_1_ and A_4_ are produced by *L. major*. The diffusion preparations were stained with Coomassie blue and dried before photographing.

Table 1 and Figure 3c list the EF sub-serotypes of the 12 Palestinian strains. Five reacted only with the anti-serotype A serum and were, therefore, serotype A strains, three being sub-serotype A_2_ and two sub-serotype A_9,_ revealed by the partial cross-reactivity disclosing partial identity of the two types of EF and indicated by the a spur forming at the junction of the two adjacent EF-antibody precipitation bands developing in the diffusion gel. The other seven reacted with, both, the anti-serotype A and the anti-serotype B sera and, therefore, produced EFs with an A and a B component, and were of the mixed sub-serotype A_9_B_4_ (Figure 4). The presence of either a sub-serotype A_2_ or a sub-serotype A_9_ component in an EF indicates that a strain is one of *L. tropica*. Also, the presence of a B_4_ component in an EF seems to indicate that the strain is one of *L. tropica*, and, in fact, the presence of a B_4_ component in an EF has, so far, always been accompanied by the presence of an A_9_ component, conferring the mixed sub-serotype A_9_B_4_ on strains producing this type of EF, in, both, this study (Table 1) and in all the other strains with the B_4_ component that have been serotyped.

#### Multilocus enzyme electrophoresis (MLEE)

Seven (LRC-L882, -L883, -L885, -L886, -L887, -L889, -L893) of the ten Palestinian strains examined by MLEE had enzyme profiles identical to those of the two Israeli reference strains (ISER/IL/98/LRC-L758 and MHOM/IL/97/P963 = LRC- L725), showing that they belonged to the zymodeme MON-137 [14]. A novel finding was that the other three strains (LRC-L884, -L890, -L891) were identical in their enzyme profiles and constituted a new zymodeme, zymodeme MON-307. Ten of the electrophoretic mobilities of the 15 enzymes in their enzyme profile were different from those of the strains in the zymodeme MON-137 (Table 2). For strains of the zymodeme MON-307, the enzyme MPI occurs as two electromorphs MPI^110^ and MPI^100^, only one of which, MPI^110^, is shared by the enzyme profile of strains of the zymodeme MON-137. This can be explained by the existence of two alleles each of which determines an isoform of the enzyme. These are identical in some cases, giving just one band, and in others not, giving two bands.

**Table 2:** **The zymodemal profiles of the strains of*Leishmania tropica*, indicating the specific differences between zymodemes encountered in Israel and the Palestinian authority**

**Enzyme**	**Country**	**MDH**	**ME**	**ICD**	**PGD**	**G6PD**	**GLUD**	**DIA**	**NP_1_**	**NP_2_**	**GOT_1_**	**GOT_2_**	**PGM**	**FH**	**MPI**	**GPI**
MON	Iraq	100	95	100	93	82	95	110	450	100	135	90	100	100	110	76
-6	Lebanon														100	
MON	Syria	112	95	100	93	82	95	110	450	100	135	90	108	110	110	76
															100	
-76																
**MON**	Palestine	**100**	**95**	**100**	**93**	**82**	**80**	**110**	**450**	**100**	**135**	**90**	**108**	**105**	**110**	**76**
															**100**	
**-307**																
MON	Israel	116	95	100	95	82	95	100	450	100	135	90	108	100	110	76
															100	
-275																
MON	Israel	112	95	100	94	82	95	100	450	100	135	90	108	100	110	76
-288															100	
MON	Israel	112	95	100	100		80	120	450	90	135	90	100	110	100	76
-54																
MON	Jordan Israel	100	100	100	98	85	80	100	460	110	140	85	108	100	110	76
-265																
**MON**	Palestine	**100**	**110**	**100**	**98**	**85**	**80**	**100**	**450**	**110**	**140**	**85**	**88**	**100**	**110**	**76**
**-137**	Jordan															
	Israel															
	Egypt															
MON	*L.aethiopica* Ethiopia	130	108	100	140	82	95	100	700	120	127	110	123	**105**	105	53
-14																
MON	*L.major* outgroup	160	88	100	122	94	200	100	400	90	110	110	118	79	150	77
-26*																

**Figure 5:**
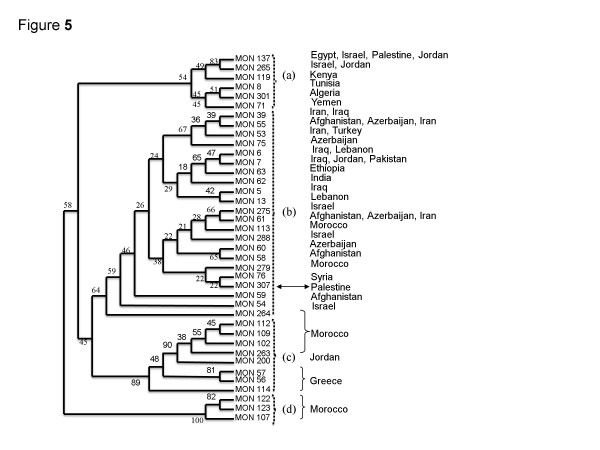
**The taxonomic relationship of the zymodemes of* Leishmania tropica.*** The dendrogram shows the geographical distribution of the known zymodemes of *L. tropica* incorporating two new zymodemes of the species, zymodemes MON-288, comprising one strain from the Negev, Israel, [14] and MON-307 (at arrow), comprising the three strains from the Jenin District. This is based on the branch encompassing the strains and zymodemes of *L. tropica* presented by Pratlong [19] in Figure 3 of their article. Unweighted Pair Group Method with Arithmetic mean (UPGMA) phenogram obtained from the matrix of presence ⁄ absence of enzyme characters. 39 zymodemes representative of the genetic diversity of *L. tropica* were analyzed. Two new zymodemes were included in the data set; zymodeme MON-288, comprising one stock from the Negev, Israel, [12] and MON-307 (arrow) representative of the three stocks from the Jenin District. For each zymodeme, the different countries of origin are indicated on the right. Bootstrap values are indicated above the branches and the dash boxes (**a**), (**b**), (**c**) and (**d**) denote different clusters.

This is modified from Schnur [14]to include the new zymodeme of *L. tropica* MON-307 and the zymodeme MON-265 associated with strains of *L. tropica* from the focus just north of the Sea of Galilee [32], but previously found associated with strains of *L. tropica* from Qadisieh in the Tafila area south-east of the Dead Sea in Jordan [36]. The enzyme profiles associated with the zymodemes MON-6, MON-76, MON-275, MON-288, MON-54 are those of other strains of *L. tropica* from the Middle East for comparison when viewing the dendrogram of the zymodemes of *L. tropica* in Figure 5. The profile representing the species *L. aethiopica* is that of the WHO reference strain *L. aethiopica* MHOM/ET/72/L100. The enzyme profile of *L. major* MHOM/IL/67/JerichoII from the Jordan Valley belonging to the zymodeme MON-26 was used as an out-group. (Note: There are three errors in the electrophoretic mobilities quoted in Table 4 in Schnur *et al*. (2004) [14]: in the profile given for MON-275, the mobility of FH should be **100** not 110; in the profile given for MON-26, the mobility of FH should be **79** not 72, and the mobility of MPI should be **150** not 50). They are corrected here.

^*^The out-group is a geographically widely distributed zymodeme of *L. major*[19].

In the reconstructed branch of the zymodemes of *L. tropica* (see under Methods for MLEE), the new zymodeme of *L. tropica*, zymodeme MON-307, fell into the sub-group (b) (Figure 5); and, within the sub-group (b) itself, the strains in zymodeme MON-307 were closest to those in the zymodeme MON-76, which were isolated in Syria. The electrophoretic mobilities of only three of the 15 enzymes, MDH, GLUD and FH, differed between the profiles of the two zymodemes (Table 2).

## Discussion

*Leishmania tropica* is a very heterogeneous species and its intraspecific micro-heterogeneity has been demonstrated by several investigators in different ways and examining various parasite constituents: EF serotyping using rabbit polyclonal anti-leishmanial sera [15] and anti-leishmanial specific mouse monoclonal antibodies [16]; ITS genotyping [9]; single strand conformation polymorphism (SSCP) of the ITS1 sequence [14,22]; DNA fingerprinting [14,22]; MLEE [18,19,23]; kDNA [12]; PPIP-PCR [24]; MLMT [3]. In the dendrogram constructed, based on MLMT data, all of the Palestinian strains of *L. tropica* fell into the sub-groups 'I: Middle East', designated A1 here, and 'II: Asia', designated B1 here (see Figure 2 for derivation of the terms A1 and B1 and their sub-groups) [3].

The three methods used in this study for the characterization of strains of *L. tropica*: EF serotyping; kDNA analysis; and MLEE, confirmed the main sub-division detected by MLMT. Two of these methods, EF serotyping and kDNA RFLP are however, much easier and quicker to perform. Furthermore, EF serotyping and MLEE revealed that the strains assigned to different genetic groups present different phenotypes which, possibly, reflect differences in leishmanial parasite – sand fly vector interrelationships.

Amplification of the kDNA minicircle sequence and RFLP analysis of the PCR products of the 12 Palestinian strains after their digestion with either *Rsa*I or *Mbo*I also identified them as strains of *L. tropica*, and enabled their separation into the kDNA Clades *Ltro-*kD1 and *Ltro-*kD2, respectively. The analysis of the kDNA RFLP profiles also exposed a further level of micro-heterogeneity and differentiation whereby three different sub-types were detected in Clade *Ltro-*kD1 and six in Clade *Ltro-*kD2 (Figure 3c). Furthermore, this micro-heterogeneity among the mini-circle kDNAs showed partial congruity with the local geographical distribution of the cases from which these strains of *L. tropica* were isolated (Table 1).

The results of EF serotyping correlated with those of the molecular biological analyses just mentioned, showing that the 12 Palestinian strains were strains of *L. tropica* of two antigenic types as indicated by the serological reactions of the strains and subsequently determined EF sub-serotypes; this exposed two serological sub-groups that coincided exactly with the two sub-groups delineated by amplification of the leishmanial kDNA minicircle sequence and RFLP analysis. This serological sub-division was based on the A component of strains' sub-serotypes: one sub-group possessing the EF sub-serotype A_2_; the other the EF sub-serotype A_9_ or A_9_B_4_ In fact, also the presence of the B_4_ component in the EF sub-serotype of some of the strains in the latter sub-group but not in other strains in this sub-group indicates a further antigenic sub-division. It is interesting to note that antiserum raised in rabbits against living promastigotes of the strain *L. donovani,* LRC-L52, according to the method of Adler [25], has never before differentiated serotype B components of EFs into B sub-serotypes except in the case of strains of *L. tropica* of the mixed sub-serotype A_9_B_4_ as shown in this study. Where enzyme profiles have also been determined, the B_4_ component seems to be associated solely with strains of *L. tropica* belonging to the zymodeme MON-137 and the B_4_ component, it seems, is always present together with the A_9_ component (Table 2 and Figure 3c).

EFs are glyco-conjugates, bearing antigenic carbohydrate moieties that confer serological specificity on the different antigenic types of leishmanial parasite, which, approximately, coincides with leishmanial species designations [16]. They are copiously produced and released by promastigotes to their environment, which is the culture medium surrounding them during culture *in vitro*[15,26]. In nature, promastigotes grow in the mid- and foregut, and in some Latin American species also the hindgut, of female sand flies where they release EF into the lumen of the vector's gut. This serves to protect the promastigotes from harmful effects of the sand fly's digestive enzymes [27]. Amastigotes also produce EF but much less so [26]. In addition to producing EF, promastigotes generate lipophosphoglycan (LPG), bearing similar and, possibly, even the same antigenic carbohydrate moieties conferring serological specificity on EFs. It is anchored in the cell membrane; though some is also released and is, supposedly, one of the antigenic components in EFs. LPG appears to promote promastigote survival in the sand fly vector by facilitating attachment to the wall of the sand fly's midgut, preventing the voiding of the promastigotes with the remnants of the digested blood meal [7,28]. LPGs extracted from the promastigotes of different leishmanial species have different molecular structures [29,30] that appear to govern leishmanial species - sand fly vector species specific attachment to the midgut wall and survival in the vector [31] as well as defining serological specificities accounting for different EF serotypes. For example, a strain of *L. tropica* from a focus just north of the Sea of Galilee, characterized as belonging to zymodeme MON-265 and EF sub-serotype A_4_, survived and grew in female sand flies of the species *Phlebotomus* (*Adlerius*) *arabicus*[7], a proven vector in that focus [32], but did not survive in female sand flies of the species *Ph.* (*Paraphlebotomus*) *sergenti*, the proven vector in other Israeli foci [14]. Conversely, the same study showed that a strain of *L. tropica* from a more southerly focus near Tiberias, producing EF of the sub-serotype A_9_B_4_ and, therefore, probably belonging to the zymodeme MON-137 (compare with Table 1) survived and grew well in females of both sand fly species. In this case, two very different vectors were involved in the transmission of the two different sub-types of *L. tropica*, supposedly, associated with ecological differences in the two habitats from which the two different sand fly species and two sub-types of *L. tropica* came. While the experimental cross-infectivity study [7] mentioned above does not impinge directly on the leishmanial strains used here, it does raise an interesting point. Since the strains of *L. tropica* of both EF sub-serotypes A_4_ and A_9_B_4_ survived in the sand fly species *Ph.* (*A.*) *arabicus*; but only the strain producing EF of the sub-serotype A_9_B_4_ survived in *Ph.* (*P.*) *sergenti*, one might assume that the lack of survival of the strain of the EF sub-serotype A_4_ in *Ph.* (*P.*) *sergenti* is a parasite-based effect rather than a sand fly vector-based one. It would be interesting to identify the sand fly vector(s) transmitting the two sub-types of the Palestinian strains of *L. tropica* described in this study, to establish if they are transmitted by the same vector or if each sub-type of *L. tropica* has its own species or even just sub-species of the same vector. However, despite the strain of *L. tropica* belonging to the zymodeme MON-265 and producing EF of the sub-serotype A_4_ and LPG like that produced by *L. major*, it did not survive and grow in female sand flies of the species *Ph. papatasi*, the proven sand fly vector of *L. major* (Svobodova, unpublished data mentioned in Svobodova [7]). It seems, therefore, that other factor(s), in addition to producing the 'correct' type of EF and LPG, is or are required for infection of female *Ph. papatasi* to succeed. Dobson [31] also came to this conclusion after completing their refined study on the effect of genetically manipulating the LPG structure of strains of *L. major* and checking their survival in their “selective” vector species, *Ph. papatasi*, and “permissive” vector species, *Ph. duboscqi*. In the study, they also engineered a strain of *L. donovani* to produce LPG of *L. major* that gave maximal survival of *L. major* in *Ph. papatasi*, but despite this manipulation the promastigotes failed to survive, indicating that another factor or factors besides the ' correct ' LPG is or are required for their survival. Since the promastigotes of the 12 Palestinian strains of *L. tropica* were of two serological/antigenic types: EF sub-serotype A_2_, congruent with zymodeme MON-307 and the minicircle DNA sequence *Ltro*-kD1, and serotype A_9_B_4_, congruent with zymodeme MON-137 and the minicircle sequence DNA *Ltro*-kD2, with each type, supposedly, also bearing a surface LPG with the same antigenic determinants that enables successful attachment in their specific natural vector, there is the possibility of two sand fly species or just sub-species transmitting the two Palestinian types of *L. tropica* in the foci of CL in the Jenin District.

Despite the introduction of many new methods for characterizing and identifying strains of *Leishmania*, MLEE is still the widely accepted standard means for differentiating leishmanial species and exposing biochemical micro-heterogenicity and interrelationships among strains, within the species [18,19,33,34]. The report given here is the first on the enzyme analysis of Palestinian strains of *L. tropica* and the zymodemes to which they belong. Ten of the 12 Palestinian strains were subjected to MLEE that also showed that the strains were strains of *L. tropica*, according to the electrophoretic mobilities of certain enzymes in their enzyme profiles, notably NP1 and GOT1. The electrophoretic mobilities of other enzymes in their enzyme profiles separated the ten strains into two sub-groups: seven belonged to the zymodeme MON-137; three to the new zymodeme MON-307, which are the reference strains of this zymodeme (Table 2). These sub-groups were fully congruent with those identified by kDNA analysis, EF serotyping and the MLMT done by Schwenkenbecher [3] (Table 1). Interestingly, the strains of *L. tropica* belonging to zymodemes MON-137 and MON-307 overlapped geographically within the limited region from where the strains came (Figure 1a and b).

Of the 15 enzymes used in formulating the enzyme profiles, one, fumarate hydratase had an electrophoretic mobility (FH^105^) different from the electrophoretic mobilities of all the known variants of FH found so far among strains of *L. tropica* examined by MLEE in the way described above (Table 2). However, FH of this mobility (FH ^105^), while being unique to the strains of *L. tropica* belonging to the zymodeme MON-307, has been found in all the strains of *L. aethiopica* analyzed by this method of MLEE ([18,19]; Table 2). The seven Palestinian strains belonging to the zymodeme MON-137 were definitely *L. tropica* and strains of *L. tropica* belonging to this zymodeme also occur in Israel [14]; the Sinai Peninsula, Egypt, [19] and Jordan [35,36]. The UPGMA dendrogram (Figure 5) indicates that strains belonging to zymodeme MON-137 have a more marginal enzyme profile than most other strains of *L. tropica* and are closer to strains of *L. tropica (syn. L. killicki*), from Tunisia in the zymodeme MON-8 and strains of *L. tropica* from Algeria, Yemen and Kenya in the zymodemes MON-301, MON-71 and MON-119, respectively, that are all found in cluster (a) in Figure 5. Pratlong [19] showed that strains of *L. tropica* belonging to the zymodemes MON-137 and MON-265, also found in Israel and Jordan [35,36], clustered more closely with one another than they did with any of the other strains falling into their '*L. tropica* complex', despite significant differences in geographical distribution, sand fly vector specificity, EF serotypes, enzyme profiles and some of their molecular biological criteria [3,7,14,32]. Correspondingly, strains of the zymodemes MON-137 and MON-265 subjected to MLMT clustered together in the sub-population I: Middle East, but those of zymodeme MON-137 belonged to the microsatellite sub-sub-groups A1a and A1b and those of zymodeme MON-265 belonged to the sub-sub-group A2b, a grouping into which no Palestinian strains fell. Combining results from MLEE and MLMT showed that the zymodeme MON-137 encompassed strains in the microsatellite sub-sub-groups A1a and A1b and zymodeme MON-307 encompassed strains in the sub-sub-groups B1a and B1b, demonstrating the greater discrimination of MLMT (Figure 2).

A similar degree of discrimination was previously shown for strains of the zymodemes of *L. infantum*, which were studied using MLMT [37] and *L. donovani,* which were studied using multilocus sequence typing (MLST) [38]. Strains with the same enzyme profile are not necessarily a genetically monolithic group. In fact, Mauricio [38] showed that enzymes of identical electrophoretic mobility can have different amino acid sequences, and that the resulting changes in molecular mass and charge can in some cases balance one another, conferring the same electrophoretic mobility, and indistinguishable zymodemal phenotypes can result from distinct genotypes.

Strains of zymodeme MON-137 seem to be restricted to the western part of the Middle East and those of zymodeme MON-307 appear unique to the northern part of the Palestinian West Bank but, on comparing enzyme profiles and microsatellite profiles of strains of *L. tropica*, those of the new zymodeme MON-307 are taxonomically closer to strains of *L. tropica* that are geographically much more distant from the foci in the Jenin District (Table 2 and sub-group (b) in Figure 2 and Figure 5). Only three enzymes had different mobilities (MDH^112^, GLUD^95^ and FH^110^) in the 15 enzyme profile of Syrian strains of zymodeme MON-76, compared with these enzymes (MDH^100^, GLUD^80^ and FH^105^) in the 15 enzyme profile of strains of zymodeme MON-307; and only three enzymes had different mobilities (GLUD^95^, PGM^100^ and FH^100^) in the 15 enzyme profile of some Lebanese and Iraqi strains, compared with these enzymes (GLUD^80^, PGM^108^ and FH^105^) in the 15 enzyme profile of strains belonging to the zymodeme MON-307. This might suggest importation of this type of *L. tropica* but it is sufficiently different from its geographically distant relatives to think otherwise.

kDNA analysis, EF serotyping and MLEE, were able to split the 12 Palestinian strains into the same two sub-groups to the same degree as could MLMT (Tables 1 and 2; Figures 2 and 3b). The strains displaying the enzyme profile associated with the zymodeme MON-307 also possessed kDNA minicircles with the sequence *Ltro*-kD1, a microsatellite profile that fell into the sub-group B1 (type II: Asia) and produced EF of the sub-serotype A_2_ while the strains displaying the enzyme profile of zymodeme MON-137 also possessed kDNA minicircles with the sequence *Ltro*-kD2, a microsatellite profile that fell into the sub-group A1 (I: Middle East) and EF of the sub-serotype A_9_ or A_9_B_4_.

The enzyme and microsatellite profiles of the strains LRC-L888 and -L1324 in kDNA Clade B are not known. However, since the nine strains LRC-L725, -L758, -L882, 883, -L885, -L886, -L887, -L889, -L893 belonging to the zymodeme MON-137 fell into the kDNA Clade B, had microsatellite profiles that fell into the sub-group A1, and either the EF serotype, A_9_ or A_9_B_4_, one can surmise that the strains LRC-L888 and -L1324 would belong to the zymodeme MON-137.

The presence of two separate sub-types of *L. tropica*, possibly, indicates two separate transmission cycles involving two separate types of phlebotomine sand fly vector. Further research should be directed at collecting potential vectors, checking for infections and isolating and characterizing leishmanial isolates from them to clarify this and to see if these leishmaniases are anthroponoses or zoonoses by searching for infected domestic, e.g., dogs, and wild, e.g., hyraxes, animals.

## Conclusions

A high degree of congruity was seen among the results of the three methods used here to characterize and differentiate Palestinian strains of *L. tropica*: EF serotyping; MLEE; and analysis of kDNA mini-circle sequences, and also with those from MLMT that was applied in a previous study [3], even though the types of criteria examined were very different from one another, having been derived from different cellular components with different intra-cellular origins within the parasites. The results from the kDNA analysis corresponded well with those of MLMT and could, possibly, replace the more complicated and labour intensive application of MLMT to leishmanial parasites from this area. The Palestinian strains that were assigned to different genetic groups also differed in their MLEE profiles and their EF types. A novel outcome of this study was the discovery of a new zymodeme, MON-307 that, at this time, seems to be unique to the northern part of the Palestinian West Bank. As two sub-types of *L. tropica* are circulating in the JD, future studies could investigate whether they are transmitted by the same sand fly species or indicate the presence of two different transmission cycles, involving separate sand fly vectors species; and whether the two different phenotypes reflect differences in the biological functions of the two parasitic sub-types, such as virulence, in different human hosts and attachment and survival in sand fly vectors.

## Competing interests

The authors declare that they have no competing interests.

## Authors' contributions

KA: conception and design of the study, involved in the collection of samples*,* extraction of DNA, PCR-RFLP, drafting the paper, analysis and interpretation of data, revising. LS: carried out all of the serotyping and its analysis, wrote his part of the Methods and also helped with the overall analysis of the results, the presentation of the data, including the figures and tables and the writing of the manuscript. GS: analysis and interpretation of data, drafting the paper and revising. AN: Providing the reference samples, revising and drafting the paper. JD and FP: carried out all of the MLEE work, wrote their part of the Methods and reviewed the manuscript, lending their expertise on the analysis of the results. CR and FB: analysed the zymodemal data of the Centre National de référence des *Leishmania,* Montpellier, and constructed the dendrogram displaying the geographical distribution of the zymodemes of *L. tropica* and their interrelationship, and were responsible for writing their part of the Methods and reviewing the manuscript and its analyses of the various data presented. SE: Revising and drafting the paper. ZA: conception and design of the study, providing the laboratory facilities, supervision of the work, analysis and interpretation of data. All authors read and approved the final version of the manuscript.
